# NTD programmes hold the key to universal health coverage and better climate and emergency responses

**DOI:** 10.1371/journal.pgph.0001738

**Published:** 2023-05-10

**Authors:** Ibrahima Socé Fall, Camilla Lavender Ducker, Anthony W. Solomon

**Affiliations:** Global Neglected Tropical Diseases Programme, World Health Organization, Geneva, Switzerland; McGill University, CANADA

Neglected tropical diseases (NTDs) inflict significant physical, mental, economic, and social harm on over one billion people worldwide, mostly in tropical and sub-tropical areas [1, 2]. Sometimes they cause death. Even when they are not fatal, however, they often lead to life-long disability, stigma and exclusion [3]. The health and social impacts of these diseases lead to increases in poverty and hunger and to reductions in children’s ability to learn. They make people less able to play a full and active part in their own lives and in the lives of their communities, contributing to a vicious circle of poverty and poor health outcomes.

Dedicated health initiatives and programmes to combat NTDs, as well as the increased integration of services and including better mapping and surveillance, have led to progress against broad development goals, such as the United Nations’ 2030 Sustainable Development Goals numbers 1, 2, 3, 6 and 10. As of December 2022, 47 countries had eliminated at least one NTD, and in the five years between 2016 and 2020, more than one billion people annually received NTD interventions [4]. These sustained efforts have reduced the global burden of disease across the NTD spectrum, bringing about notable, much-needed gains for people whose lives might otherwise have been devastated.

This is, of course, good news; the health system-strengthening initiatives and the development of an extensive, widely skilled health force on the ground that enabled these gains mean that places with otherwise threadbare or non-functional health services have received a minimum level of health provision.

NTDs, in this context, provide us with crucial indicators [5]. Their collaborative approach and community-level implementation, as well as their ability to reach remote populations and to do so equitably, present a valuable, reproducible model for health and development programmes more generally [6].

We propose that some of the most pressing issues in global health today, such as climate change, the drive for Universal Health Coverage, and better detection of new and emerging health emergencies, can be addressed by leveraging the power, reach and know-how of those who plan and deliver NTD programmes.

Evidence on the health consequences of climate change is beginning to accumulate [7]. WHO’s statements have shifted in proportion. Where previously it was considered “a significant and emerging threat to public health”, climate change is now described as “the single biggest health threat facing humanity” [8] Climate change affects and will affect human, animal and environmental health both directly (by increasing morbidity and mortality, and by weakening of quality health care provision as resources become more and more stretched) and indirectly, as individuals, households and communities see their ability to resist changing biological, epidemiological, and economic realities dramatically decline.

The evidence suggests too that these impacts are being and will be disproportionately felt by poor people [9]. Further mapping work may well be needed, but it can be surmised that the overlap of these communities with those currently experiencing the highest burdens of NTDs will be significant.

On the macro level, climate change appears set to upend the distribution and magnitude of NTD prevalence and incidence as environmental conditions alter the dynamics of pathogen-vector-host relationships. While it is difficult to generalise about a diverse set of 20 diseases and disease groups, each with its own complexities and specificities, increases in the burden of many NTDs are predicted, both in areas where transmission is already intense as well as in areas that are not currently affected [10].

Needless to say, this will compound the negative health and well-being impacts on populations at risk from NTDs and make efforts to control, eliminate and eradicate them far more complicated. Effects on animal health promise to be just as significant and, with more than 70% of rural livelihoods globally dependent on the farming of livestock, this constitutes a significant vulnerability [11].

All of this underlines the urgency of a combined, collaborative, integrated, intelligent and equitable approach to the multifaceted problems our world seems certain to face in the coming years. The One Health approach, whereby human and animal health, as well as the physical and social environment, are assessed, analysed and planned for in a holistic continuum, is an example of one such attempt to join the dots.

It is also an approach that the NTD world already employs to a large extent. The interrelated nature of NTDs, many of which are zoonotic, vector-borne, and/or directly related to human activities in the environment, has meant that planning for their control and treatment, on the animal-human-environmental interface, already takes place.

Increases in human populations, as well as increased population movement–in part due to climate change–are altering human-animal interactions, and may lead to increased human infections and co-infections, as well as the emergence of novel diseases [12]. Parasitic hybridization can occur during co-infection, complicating disease control measures, while the emergence of new diseases can have global repercussions, amply demonstrated by the COVID-19 pandemic [13]. And while there was much discussion of the probable zoonotic origins of COVID-19, it has been remarkable how comparatively little discussion there has been about the need to monitor and manage animal host communities, even in the face of reliable predictions of a rise in new infectious human diseases of zoonotic origin [14].

In this context, existing NTD intervention models offer a way forward. A cross-cutting NTD/One Health approach, as described in the 2021–2030 NTD road map, could deliver paradigm-shifting gains both now and in the future [7].

There are important lessons we can harness to inform our understanding of how to prevent and eliminate diseases, as well as guide our attempts to head off diseases that could emerge in the future. One clear example is the necessary drive towards Universal Health Coverage, as we as a global health community seek to ensure equitable and effective basic standards of healthcare for people everywhere, based on need rather than ability to pay. This is a key priority of the UN’s Sustainable Development Goals.

NTD control measures have repeatedly shown that it is possible to serve the most vulnerable and marginalised people in the global community [15]. The task now–and it will only become more urgent–is to use, mainstream and augment these platforms to turn disease-specific interventions into guarantors of the basic but wide-ranging services which will improve community health across the board.
